# A DFT Computational Study of Type-I Clathrates A_8_Sn_46−x_ (A = Cs or NH_4_, x = 0 or 2)

**DOI:** 10.3390/ma17184595

**Published:** 2024-09-19

**Authors:** Nikolaos Kelaidis, Emmanuel Klontzas, Andreas Kaltzoglou

**Affiliations:** Theoretical and Physical Chemistry Institute, National Hellenic Research Foundation, 11635 Athens, Greece; nkelaidis@eie.gr (N.K.); klontzas@eie.gr (E.K.)

**Keywords:** clathrates, host-guest interactions, Zintl-Klemm concept, DFT calculations, phonon scattering

## Abstract

Semiconducting clathrates have attracted considerable interest in the field of thermoelectric materials. We report here a computational study on the crystal structure, the enthalpy of formation, and the physical properties of the following type-I clathrates: (a) experimentally studied Cs_8_Sn_44_ and hypothetical Cs_8_Sn_46_ and (b) hypothetical (NH_4_)_8_Sn_46−x_ (x = 0 or 2). The ab initio VASP calculations for the nominal stoichiometries include the geometry optimization of the initial structural models, enthalpies of formation, and the electronic and phonon density of states. Comparison of the chemical bonding of the structural models is performed via the electron localization function. The results show that the presence and distribution of defects in the Sn framework for both Cs_8_Sn_46−x_ and (NH_4_)_8_Sn_46−x_ systems significantly alters the formation energy and its electrical properties, ranging from metallic to semiconducting behavior. In particular, one defect per six-membered Sn ring in a 3D spiro-network is the thermodynamically preferred configuration that results in the Cs_8_Sn_44_ and (NH_4_)_8_Sn_44_ stoichiometries with narrow-band gap semiconducting behavior. Moreover, the rotation of the ammonium cation in the polyhedral cavities is an interesting feature that may promote the use of ammonium or other small molecular cations as guests in clathrates for thermoelectric applications; this is due to the decrease in the lattice thermal conductivity.

## 1. Introduction

**Figure 1:**
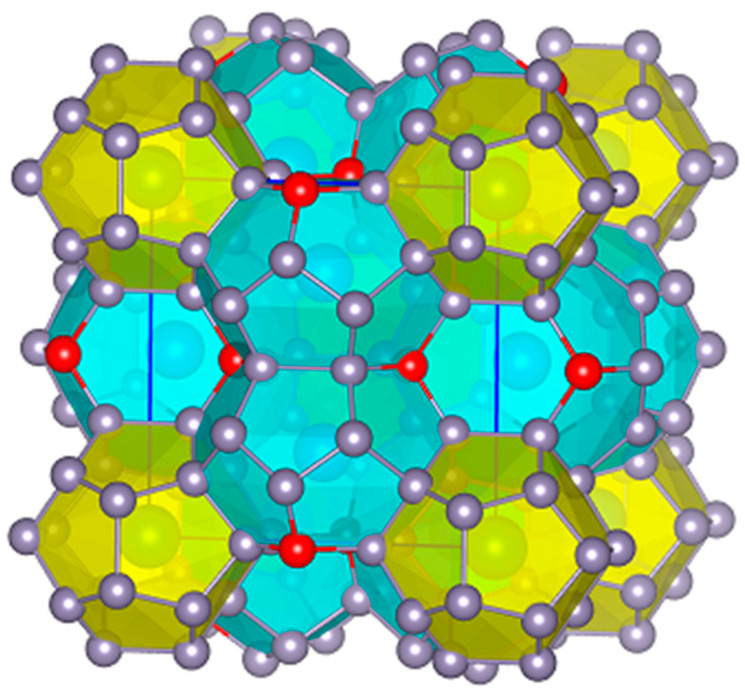
Crystal structure of the ideal clathrate-I Cs_8_Sn_46_. Columns of tkad (light blue) run orthogonally to one another and the voids are filled with pdod (yellow). Cs atoms reside in the center of the polyhedra. Sn atoms that bridge the pdod are in red to denote their experimentally determined partial occupancy, which leads to the actual Cs_8_Sn_44_ composition. Unit cell edges are shown in blue.

“Semiconducting” or “intermetallic“ clathrates are a distinct class of inclusion compounds. In contrast to clathrate hydrates or organic clathrates that incorporate water or organic molecules as host framework, respectively, semiconducting clathrates mainly incorporate moderately electronegative (p-block and d-block) elements as the host framework (atoms X) and highly electropositive or electronegative elements as guests (atoms A) [1,2]. Various clathrate structural types are known that combining polyhedra into a 3D network, with type-I A_8_X_46_ (Figure 1) and type-II A_24_X_136_ as the most common. The covalently, four-bonded X atoms form the following polyhedra: (a) pentagonal dodecahedron (pdod, [5^12^]) with 20 vertices, (b) tetrakaidecahedron (tkad, [5^12^6^2^]) with 24 vertices, (c) pentakaidecahedron (pkad, [5^12^6^3^]) with 26 vertices, and (d) hexakaidecahedron (hkad, [5^12^6^4^]) with 28 vertices. The symbols in brackets denote the number of pentagonal and hexagonal faces of the polyhedron. The formation of different clathrate types depends mainly on the guest size. These compounds are often non-stoichiometric on either the X or A site, and mixed occupancies between different atom types occur. Even “empty” clathrates exist as thermodynamically metastable allotropic forms of host elements, e.g., type-II Ge_136_ [3]. In general, these open framework structures show a thermodynamic tendency towards dense states. The guest atom or ion must be small enough to “fit” in the rigid covalent cage without stereochemical hindrance, but, on the other hand, should not be too small and bound very loosely or even “escape” from it. In terms of cavity size, the small pdod made of Si atoms (e.g., in type-I Ba_8_Si_46_) has a d(Si-Si)_max_ of ca. 6.8 Å, whereas the large hkad made of Sn atoms (e.g., in type-II K_0.9_Ba_15.1_Ga_31_Sn_105_) has a d(Sn-Sn)_max_ of ca. 9.1 Å. In this context, clathrate compounds do not usually exist with small cations such as A = H, Li Na, Be, and Mg but do exist with A = K, Rb, Cs, Ca, Sr, and Ba [4].

With regard to the physical properties, the majority of clathrates are either intrinsic or extrinsic semiconductors and their stoichiometry follows the electron-counting rules of the Zintl–Klemm formalism [5] for polyanionic (more common) and polycationic (less common) frameworks. The underoccupancy of crystallographic sites can be described as Zintl defects [6,7]. Overall, many classes of compounds that follow the Zintl–Klemm concept have been considered as promising candidates for thermoelectric applications [8,9]. In particular, the thermoelectric efficiency of the clathrates has been proposed by G. Slack in terms of the Phonon Glass-Electron Crystal concept (PGEC) [10]. The basic hypothesis is that a semiconducting host framework has high Seebeck coefficient and electrical conductivity (like in crystals), while the extreme thermal motion—“rattling”–of the loosely-bound guest atoms efficiently scatters the heat-carrying phonons and reduces the lattice contribution of thermal conductivity (like in glasses) [11,12,13,14]. Moreover, small molecular cations, such as methylammonium and formamidinium, exhibit an off-centering from the framework cavity, which hinders phonon propagation, hence reducing the lattice thermal conductivity. This effect was first seen in halide perovskite compounds for application in state-of-the-art third-generation solar cells [15].

With regard to A_8_Sn_46−x_ system, it is well established that the Sn framework contains two defects in order to fulfill the Zintl–Klemm concept according to the electron count (Cs^+^)_8_(3b-Sn^–^)_8_(4b-Sn^0^)_36_ (Figure 1). The tin clathrates were originally found to crystallize in the cubic space group *Pm-3n* and the partially occupied positions were distributed along a 4_2_ screw axis [16]. These framework sites are part of six-membered rings of the tkad and do not belong to the pdod. A Mulliken population analysis based on topological charge stabilization showed that the 6*c* framework sites (6 out of 46 for the space group *Pm*-3*n*, No. 223) possess the lowest electron density and therefore it is easier to remove a Sn atom from there than from any other position [17]. Other theoretical studies for Cs_8_Sn_44_ [18] indicated that one vacancy per six-membered ring is energetically favored over two vacancies per ring. This implies that the compound may exhibit an even higher ordering of the defects. Such a superstructure was experimentally established for Rb_8_Sn_44_ [19], which crystallizes at room temperature with a 2 × 2 × 2 unit cell in the space group *Ia-3d* (No. 230); the partially occupied sites (3 out of 46) are distributed along a 4_1_ screw axis. The same defect distribution and space group are found for the type-I clathrate Ba_8_Ge_43_, where the three Ge vacancies are fully ordered after annealing and fast cooling of the material [6]. Eventually, the conditions under which the two modifications of Rb_8_Sn_44_ and Cs_8_Sn_44_ exist were clarified based on an enantiotropic, order-disorder phase transition at 353 K and 363 K, respectively [20,21]. In particular, below the transition temperature, the high-order, lower-symmetry (space group *Ia*-3*d*) occurs, whereas above the transition temperature, the low-order, high-symmetry configuration (space group *Pm*-3*n*) occurs. The thermoelectric properties (electrical resistivity, thermal conductivity, and Seebeck coefficient) of both Rb_8_Sn_44_ and Cs_8_Sn_44_ are significantly affected by the phase transition [21,22,23]. Moreover, multitemperature Rietveld refinements in Rb_8_Sn_44_ have shown strong dynamic disorder effect of the Rb cation, as it fades almost linearly to zero with decreasing temperature to 0 K [21].

From a practical point of view, the synthesis of the A_8_Sn_44_ (A = Rb, Cs) compounds can only be performed in very inert conditions, namely in Nb or Ta crucibles and under high-purity Argon atmosphere, due to the reducing and pyrophoric nature of the alkali metals. This limits the materials preparation to small quantities (*ca.* 1 g per batch) and has a high cost. Here, we perform a computational study on the exact distribution of defects in Cs_8_Sn_44_ and we also study, for the first time, the incorporation of the ammonium cation in type-I Sn-based clathrates. On one hand, the ammonium cation is very light (M_r_(NH_4_^+^) = 18.04) compared to other commonly used cations, such as Cs (A_r_(Cs^+^) = 132.91). On the other hand, it can rotate in the center of the polyhedra cavities. In terms of ‘effective ionic radius’, the ammonium cation equals to ca. 1.5 Å [24,25], which is similar to the ionic radius of Cs at 1.7 Å. This combination of low weight and large size of the guest species may affect the phonon scattering of the system and ultimately reduce the lattice contribution to the thermal conductivity of the clathrate materials.

## 2. Materials and Methods

### 2.1. DFT Calculations

**Figure 2:**
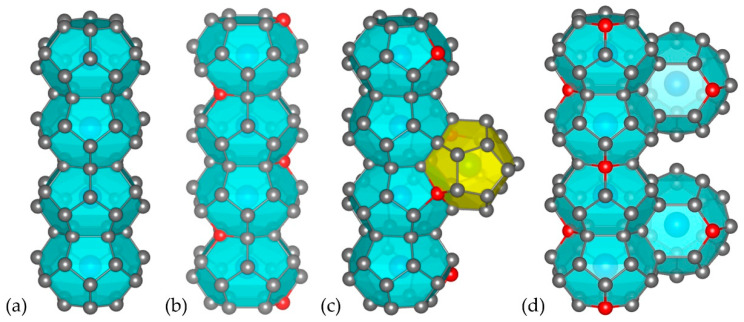
Initial structural models: (**a**) Cs_8_Sn_46_ without framework defects, (**b**) model A, (**c**) model B, and (**d**) model C for Cs_8_Sn_44_ with different distribution of the defect sites (red spheres).

The preparation of all structural models for the DFT calculations as well as the illustration of the crystal structures and the electron localization function plots (see Section 2.3) was performed using the version 3.9 VESTA software [26]. For Cs_8_Sn_46−x_, the experimentally determined structure for Cs_8_Sn_44_ at room temperature from synchrotron diffraction data [21] was used as starting point. Symmetry reduction from space group *Ia*-3*d* (No. 230) to space group *P*1 (No. 1) was performed to avoid any underoccupied Sn sites and the following four models were studied: (a) Cs_8_Sn_46_ with no defects, where the atomic coordinates are equal to the type-I clathrate archetype symmetry (space group *Pm*-3*n*, No. 223), (b) Cs_8_Sn_44_ model A, where the defect sites are located in neighboring six-membered Sn rings (4 Sn atoms in between) and form a non-crystallographic helical axis (similar to 2_1_) across the tkad chains in one of the three crystal axes (there are only half the defect sites in the other two crystal axes), (c) Cs_8_Sn_44_ model B, where the defect sites are located in five-membered rings (two Sn atoms in between) of both pdod and tkad in one of the three crystal axes, and (d) Cs_8_Sn_44_ model C, where the defects are located in six-membered Sn rings of the tkad (three Sn atoms in between) without forming a helical axis as in model A, but forming a spiro-network between interconnected tkad in all three crystal axes (Figure 2).

**Figure 3:**
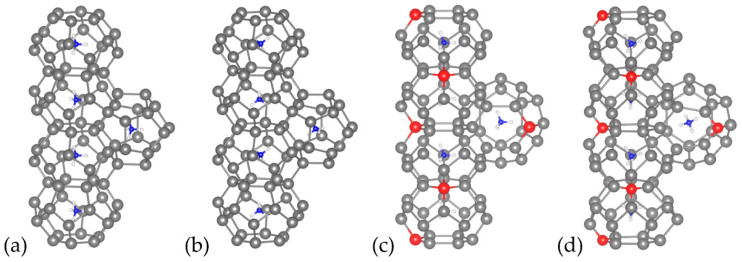
Structural models ammonium-containing clathrates: (**a**) before and (**b**) after relaxation for (NH_4_)_8_Sn_46_, (**c**) before and (**d**) after relaxation for (NH_4_)_8_Sn_44_ (model C). Red spheres indicate vacant sites within a tkad.

For (NH_4_)_8_Sn_46−x_, the same four models were used as in the case of Cs_8_Sn_46−x_ with regard to the framework atoms. As for the ammonium cation, its initial coordinates were chosen to form a regular tetrahedron (as in the NH_4_Cl crystal structure [27]) where the N atom replaces the Cs atom. The orientation of the ammonium cation in the pdod is such that the N–H–Sn angles of the closest neighbors are in the range 110–160° and no 5-fold rotational axis of the pdod coincides with a 3-fold rotational axis of the ammonium cation. The orientation of the ammonium cation in the tkad is such that a 3-fold rotational axis of the ammonium cation coincides with the 6-fold rotational axis of the tkad, and this results in two sets of N–H–Sn angles at 125° (for the six-membered rings) and 158° (for the five-membered rings) (Figure 3a).

The DFT calculations were performed on the Vienna Ab initio Simulation Package (VASP, vs. 5.4.4.) [28,29,30], which employs the plane-wave pseudopotential method. We applied the Perbew–Burke–Ernzerhof functional revised for solids (PBESOL) in order to describe the exchange-correlation interactions and we utilized the soft pseudopotentials [31,32] provided by VASP. The cut-off energy was set at 520 eV and a k-point grid of 4 × 4 × 4 was applied for the sampling of the Brillouin zone after convergence tests. The self-consistent field (SCF) criterium for our calculations is set at 1.0 × 10 ^−6^ eV/atom for the energy tolerance and 0.02 eV/Å for the force tolerance. The conjugate gradient algorithm was used for the geometry optimization of the compounds and all degrees of freedom (positions of ions, cell volume, and shape) were allowed to change. The formation energy of a compound is calculated by calculating the total energies of the compounds and their constituent elements using the formula:

(1) $$\Delta H_{f}=E_{compound}-\sum_{i}n_{i}\cdot \mu_{i}$$

where *Ε*_compound_ is the total energy calculated by DFT and *μ*_i_ the chemical potential of each element, calculated by DFT by calculating total energy (per atom) in its standard, most stable state. The Sn vacancy formation energy of compound A_8_Sn_44_ is also calculated (per pair of Sn atoms) by employing the following formula, where error correction terms have been omitted:

(2) $$E_{V_{Sn}}{=E}_{A_{8}{Sn}_{44}}-(E_{A_{8}{Sn}_{46}}-2\mu \;E_{Sn})$$

### 2.2. Electronic Properties Calculation Method

To gain an understanding of the electronic structure of the Cs and ammonium-containing clathrates, we calculate the band structures, density of states (DOS), and electron localization function (ELF) of the structural models under investigation. For the DOS calculations, a denser Monkhorst–Pack grid of 8 × 8 × 8 was applied. The band structure was calculated along the high symmetry points in the Brillouin zone. As for the geometry optimization, we employed the modified for solids Perdew–Burke–Ernzerhof exchange-correlation functional within the Generalized Gradient Approximation (GGA-PBEsol). Band structure and DOS plots are drawn with the SUMO software [33].

### 2.3. Electron Localization Function Method

The Electron Localization Function (ELF) was introduced by Becke and Edgcombe [34] in 1990 to provide additional insight into the nature of bonding between atoms and the charge distribution in the system. It is a dimensionless indicator of the localization of electrons, correlated to the probability density of a same-spin electron pair, and ranges from 0 to 1. The regions with high ELF values that are close to unity are regions of high electron localization, usually an indication of a strong covalent bond. Regions of lower ELF are regions of lower electron localization and can be found in ionic bonds. The value of 0.5 is of a free electron like delocalized gas, and lower values denote a minimal electron density concentration [35,36]. An excellent review paper on this and examples of its applications can be found in reference [37].

### 2.4. Phonon Calculations Method

Phonons were calculated by DFT using the finite displacement method (FDM) with the aid of the Phonopy [38,39] software. Phonopy introduces a systematic set of atomic displacements in the equilibrium structure, and DFT calculations were performed on each displaced structure to obtain the forces on the atoms. After the individual DFT calculations were completed, Phonopy was used to compute the force constants and the vibrational properties and stability of the material. For these calculations, the optimized structures were used. Due to the size of our unit cell of our models and the number of atoms, no supercell was used. The DFT conditions are kept the same with the geometry optimization; the GGA-PBEsol was applied, with a cutoff energy of 520 eV and a 4 × 4 × 4 Monkhorst-Pack k-point grid, with the exception of the energy convergence criterion, which was set to 1 × 10^−8^ eV.

## 3. Results

### 3.1. Structural Optimization and Calculation of Formation Enthalpies

Structural optimizations were performed for all structural models (Table 1). The selection of the PBEsol functional with respect to other functionals (LDA, PBE) was based on a better agreement with experimental results for the case of the experimentally available Cs- compounds, namely Cs_8_Sn_44_, consistent with the findings in Ref. [7], where it is also observed that PBEsol leads to more accurate vacancy formation energies in Si/Ge based clathrates. The calculated vacancy formation energies of the Cs_8_Sn_44_ and (NH_4_)_8_Sn_44_ configurations are related to the stability of Sn vacancies within the lattice. Model A and C exhibit negative vacancy formation energies (energies shown are for the formation of two V_Sn_ per cell), suggesting that these structures are energetically favorable, whereas model B shows a slightly positive vacancy formation energy, indicating a less favorable configuration. The A_8_Sn_46_ models are more stable than the corresponding B models, but significantly less stable than A and C models, the latter being the most stable configuration overall.

The Sn vacancies cause the neighboring Sn atoms to move in order to reduce the strain and charge imbalances induced by the defects and minimize the total energy of the system. This is in agreement with the crystallographic [20] and the spectroscopic (Sn Moessbauer [21] and multi-temperature Raman [40]) results for A_8_Sn_44_, excluding the possibility of having two defects within a six-membered ring even for the high-temperature modification (space group *Pm-3n*). Such a case would accumulate large negative charge between the bonding Sn atoms (the remaining four of the defect hexagon), which renders it unstable (similar to our model B). The higher thermodynamic stability of model C over model A is attributed to the more isotropic distribution of the defects in the unit cell, as model A accumulates higher negative charge in only one of the crystal axes. Overall, no other possibility for a distance greater than four atoms between the defects is available for the type-I A_8_M_44_ clathrates. In principle, the distance between two defects (or two atoms) within a cubic cell cannot exceed (3^0.5^ × *a*)/2 = 10.4 Å.

With regard to the displacement of the guest atoms from the center of the polyhedra, for Cs_8_Sn_44_ model C, Cs in pdod is displaced by 0.029 Å, whereas in the tkad it is displaced by 0.035 Å. This small displacement for the Cs cation is expected due to its relatively large size. For reference, a similar study on the dislocation or even migration of Li and Na guests from the center of the polyhedra in type-I clathrates showed much smaller mobility for Na cations compared to Li cations due to the smaller ionic radius of the latter [41]. In (NH_4_)_8_Sn_44_ model C, the deviation of the N atoms from the center of the pdod is 0.02 Å and 0.17 Å from the center of the tkad. This increase in the off-centering in the tkad compared to the pdod for both compounds is attributed to the asymmetry of the tkad imposed by the presence of one defect site, which attracts the cationic guest due to its opposite charge. In contrast, the pdod remains almost regular and neutral in terms of Sn charge. With regard to the guest-host interactions in the (NH_4_)_8_Sn_46−x_ system, the nearest Sn–H distances are at ca. 2.9 Å for (NH_4_)_8_Sn_46_ and at ca. 2.75 Å for (NH_4_)_8_Sn_44_ model C, which renders the interactions mainly ionic, rather than covalent (see Section 3.2.2 and Section 3.3 for more details). The Sn–Sn distances range from 2.80 Å (4-bonded) to 2.99 Å (3-bonded) for (NH_4_)_8_Sn_44_ model C. This elongation of the Sn–Sn bonds next to the defect sites is clearly visible in Figure 3d. With regard to the orientation of the ammonium group within the pdod and the tkad, significant differences are observed both before and after the structure relaxation as the tetrahedron rotates significantly. For model C after relaxation, even though there is no direct dual relationship between the regular dodecahedron and the tetrahedron (both are Platonic solids) [42], two neighboring ammonium group within a column of tkad exhibit a symmetry similar to the reflection plane along with a ca. 6 ° rotation.

### 3.2. Electronic Properties

#### 3.2.1. Band-Structure and Density of States for Cs_8_Sn_46−x_

**Figure 4:**
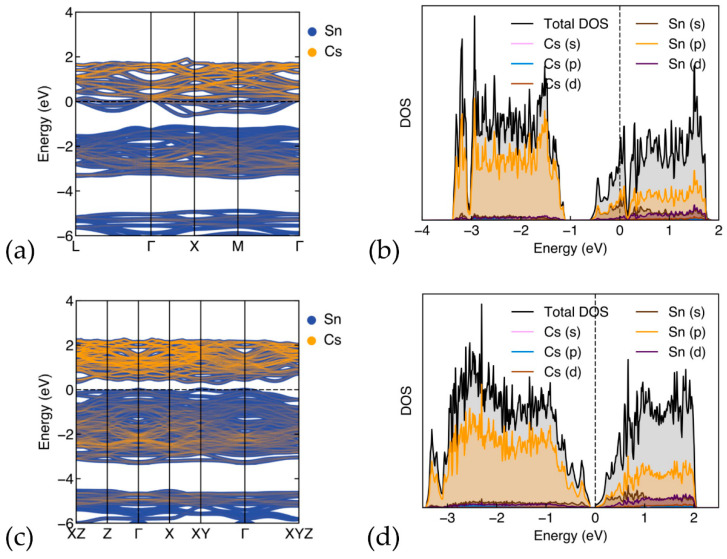
(**a**) Band structure and (**b**) DOS for Cs_8_Sn_46_, (**c**) Band structure and (**d**) DOS for Cs_8_Sn_44_ (model C). Fermi level is set at zero.

We have calculated the band structures and DOS of the optimized Cs_8_Sn_46_ and C_8_Sn_44_ (model C) (Figure 4). In Figure 4a, the band-structure of Cs_8_Sn_46_ is shown, plotted along the k point path of L(0.5,0.5,0.5), Γ(0,0,0), Χ(0.5,0,0), Μ(0.5,0.5,0), and Γ. The metallic structure of Cs_8_Sn_46_ is evident as the Fermi level (dashed line) is in the conduction band, which is in alignment with previous studies [12]. The partial density of states (Figure 4b) of Cs_8_Sn_46_ illustrates the contribution of each element to the total DOS. The total DOS is dominated by the Sn atoms, while the contribution of Cs atoms is significantly smaller. Both atoms contribute with the *p* orbitals above and below the pseudo-gap, while the d orbitals of Cs are more prominent above the pseudo-gap. There are few overlapping peaks between Cs and Sn, with some small peaks just above the Fermi level and deep in the valence band around −3 eV. This indicates minimal hybridization between Cs and Sn orbitals, suggesting that the Cs-Sn bond is not strongly covalent, leaning more towards an ionic character. The Cs atoms can act as electron donors, contributing to the conductivity, which is also evident from the Cs contribution in the band structure (Figure 4a).

The band structure of the defect Cs_8_Sn_44_ (model C) is shown in Figure 4c. The band structure is plotted along the path of high symmetry points of the Brillouin zone XZ(0.5,0,0.5), Z(0,0,0.5), Γ(0,0,0), X(0.5,0,0), XY(0.5,0.5,0), and Γ. The band structure shows a semiconducting character for this structure, with the presence of a band gap calculated at 0.28 eV. The band structure also shows that this material is an indirect band gap, where the valence band maximum and the conduction band minimum appears at XY and between XY and Γ respectively. In this work, we will be presenting model C as it is calculated to be the most energetically stable. Model A has similar electronic properties (shape of band structure and DOS) but shows an even lower band gap. Examining the partial DOS calculations of this structure (Figure 4d), we observe similarities with the Cs_8_Sn_46_ structure. Here, the main contributions arise from the Sn atoms, with Sn (p) orbitals being more prominent. Nevertheless, we observe a flatter (broader) distribution of states along the energy range than in the case of Cs_8_Sn_44_, without significant peaks.

There is an increased correlation between the Sn (p) and Cs (p and d) orbitals and there appears to be a greater hybridization in Cs_8_Sn_44_ compared to Cs_8_Sn_46_. Despite this, the overall picture is not that different, with even fewer peaks present, leading to a weak or negligible covalent interaction between Cs and Sn and pointing towards an ionic character of the Cs-Sn bond. Additionally, the bands of Cs_8_Sn_44_ are flatter (broader), which indicate less dispersion of electronic states and different electronic properties, such as reduced metallic character or changes in electrical conductivity, which is consistent with the fact that calculations predict this structure to be a semiconductor [43].

#### 3.2.2. Band-Structure and Density of States for (ΝH_4_)_8_Sn_46−x_

**Figure 5:**
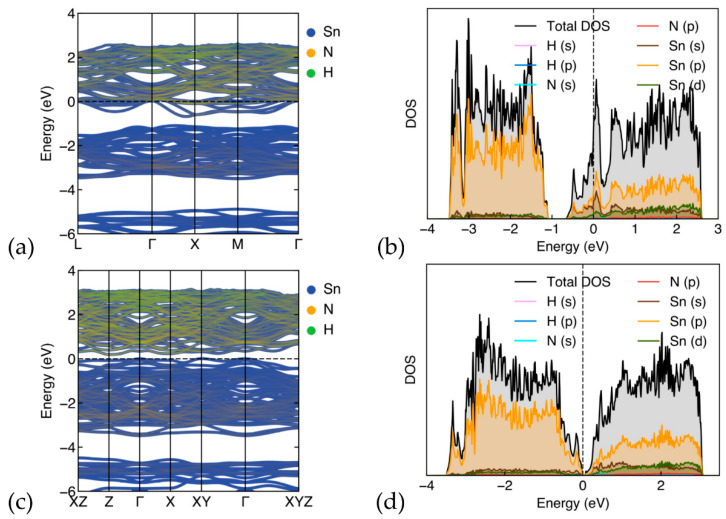
(**a**) Band structure and (**b**) DOS for (NH_4_)_8_Sn_46_, (**c**) Band structure and (**d**) DOS for (NH_4_)_8_Sn_44_ (model C). Fermi level is set at zero.

In Figure 5, the band structure and density-of-states of (ΝH_4_)_8_Sn_46−x_ (x = 0,2) are shown. In Figure 5a, the calculated band structure of (ΝH_4_)_8_Sn_46_ is presented. As in the case with Cs guest atoms, the band structure reveals a metallic character, with the Fermi energy level situated in the conduction band. In Figure 5b, the total and partial DOS are shown for this structure. We observe a similar picture to that of the Cs_8_Sn_46_ structure, mainly in the sense the DOS is dominated by framework atoms, with the Sn (p) orbitals being the most prominent, while the guest atoms, N and H, contribute less than 2% of the total DOS. Both nitrogen s and p states show small and almost equal contributions, with a peak at the Fermi level. Hydrogen s and p states display minor contributions and are more prominent in the conduction band region. The are no significant overlapping peaks in the PDOS for Sn and N or H below the Fermi level, except for the peaks at 3 eV below the Fermi level and a peak at the Fermi level. When examining the band structure of (ΝH_4_)_8_Sn_44_, we observe semiconducting behavior with a small band gap of approx. 0.18 eV and the Fermi level at the top of the valence band. Similar to the case of Cs_8_Sn_44_, this material is an indirect band gap, where the valence band maximum and the conduction band minimum appears at XY and Z, respectively. The DOS of (ΝH_4_)_8_Sn_44_ are shown in Figure 5d. In comparison with the DOS of (ΝH_4_)_8_Sn_46_, we observe a flatter image with less prominent peaks. As in the case of Cs_8_Sn_46−x_, we observe a non-covalent character for guest–host interactions and the transition from metal to semiconductor when x changes from 0 to 2. The similarities between the Sn based clathrates with Cs and NH_4_ clathrates are encouraging for the potential thermoelectric properties of the latter. Further details on the partial DOS for both A_8_Sn_46_ and (ΝH_4_)_8_Sn_44_ (model C) (A = Cs, ΝH_4_) are found in Supplementary Materials (Figures S1–S4).

#### 3.2.3. Electron Localization Function

**Figure 6:**
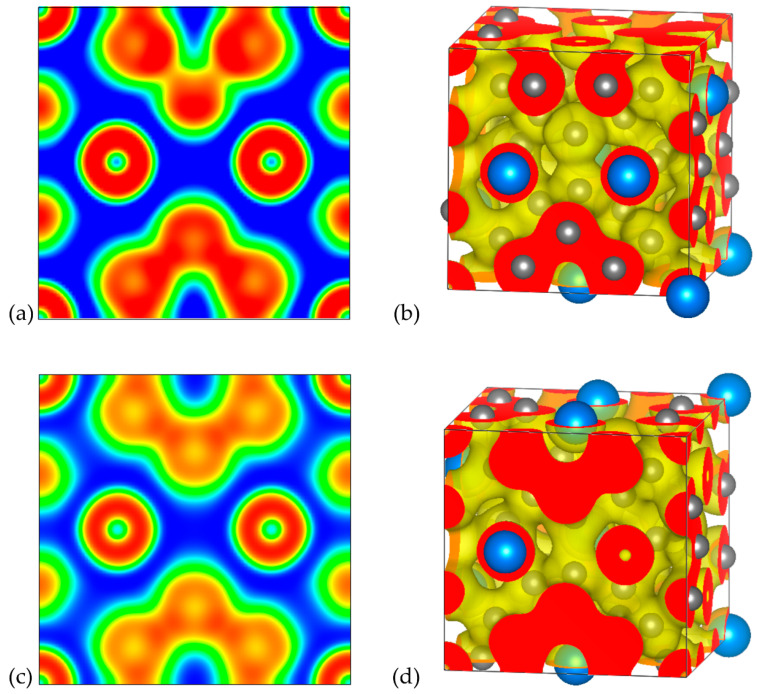
Electron localization function plots for Cs_8_Sn_44_ (**a**) 2D ELF map of the (001) plane isosurface (n = 0.01) and (**b**) 3D ELF map isosurface (n = 0.5) for model A, (**c**) 2D ELF map of the (001) plane isosurface (n = 0.01) and (**d**) 3D ELF map isosurface (n = 0.5) for model C. The colors blue, green and red refer to ELF values of 0, 0.5 and 1, respectively.

**Figure 7:**
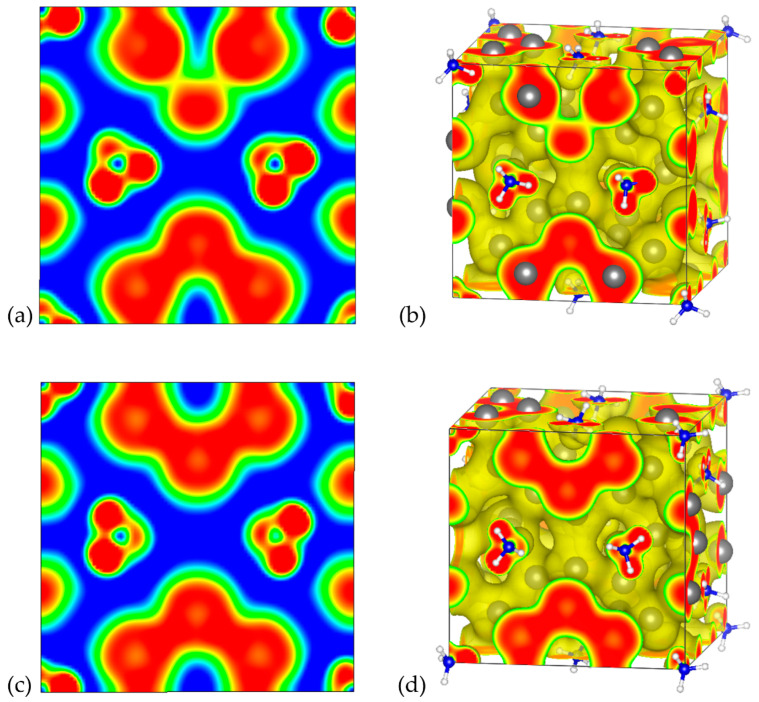
Electron localization function plots for (NH_4_)_8_Sn_44_. (**a**) 2D ELF map of the (001) plane isosurface (n = 0.01) and (**b**) 3D ELF map isosurface (n = 0.5) for the model A, (**c**) 2D ELF map of the (001) plane isosurface (n = 0.01) and (**d**) 3D ELF map isosurface (n = 0.5) for model C. The colors blue, green and red refer to ELF values of 0, 0.5 and 1, respectively.

In order to provide further insights regarding the chemical bonding of the examined systems, ELF analysis was conducted. The corresponding 2D and 3D ELF plots for A_8_Sn_44_ (A = Cs, NH_4_) are illustrated in Figure 6 and Figure 7, respectively. We focused on the analysis of the A_8_Sn_44_ structures since they are more interesting due to the Sn vacancies in their framework. The ELF is dimensionless and takes values in the range of 0 and 1 depending on the magnitude of the localization of electrons. For ELF = 1 (red), the electrons are strongly localized, while values 0.5 (green) and 0 (blue) define regions with delocalized electrons and low electron density, respectively. The ELF analysis for the Cs_8_Sn_44_ clathrate is presented in Figure 6. In Figure 6a, we present a 2D ELF map along the (001) plane of model A, along with the corresponding 3D ELF map (Figure 6b).

In the case of the three Sn atoms belonging to a six-membered ring in the bottom of the 2D map, it can be noticed that there is a strong electron localization between the Sn atoms, confirming the covalent nature of the bonds between Sn atoms. Each of these Sn atoms forms four covalent bonds with their neighboring Sn atoms in the framework. Interestingly, in the case of the Sn atoms belonging to a hypothetical six-membered ring with a missing Sn atom, as depicted in the opposite site of the 2D map, there is a region with strong electron localization, which is located in the position of vacancy and can be attributed to the formation of electron pairs from the neighboring undercoordinated Sn atoms. The ELF value in the region between the Cs guest atoms and the Sn atoms of the clathrate framework is very low, confirming the ionic nature of the interaction between the guest atoms and the host material. We have also plotted the corresponding 2D and 3D ELF maps for the model C of Cs_8_Sn_44_ (Figure 6c,d). In contrast to the 2D ELF map of model A, it can be observed that identical ELF values were found for the region between the Sn atoms forming the half of six-membered rings, since the (001) plane of the model C contain completed six-membered rings without Sn vacancies.

The 2D and 3D ELF plots for (NH_4_)_8_Sn_44_ for models A and C are illustrated in Figure 7. The 2D ELF maps shown in Figure 7a,c correspond to the same (001) plane as for Cs clathrates. Qualitatively, the obtained 2D ELF maps for the corresponding models for Cs and NH_4_ clathrates share the same features regarding the regions of electron localization in the neighbor of Sn atoms and the ionic character of the interaction between guest cations and the host framework (blue region around NH_4_ cations). High values of ELF are observed in NH_4_^+^, indicating the N-H covalent bonds existing within the guest cation. A slight distinction in the 2D ELF maps between model A for Cs and NH_4_ clathrates can be observed if we compare the illustrations in Figure 6a and Figure 7a. In the case of the NH_4_ clathrate, lower ELF values are observed in the region of the Sn vacancy, which can be attributed to the weaker ability of charge transfer from the ammonium cation with respect to Cs.

### 3.3. Phonon Calculations

**Figure 8:**
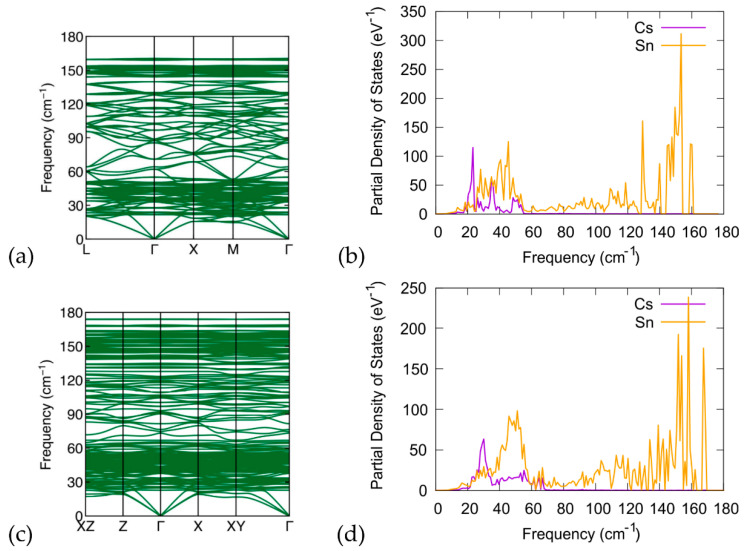
(**a**) Calculated phonon band structure along the L-Γ-Χ-Μ-Γ path for the Cs_8_Sn_46_, (**b**) partial phonon density of states for Cs and Sn of the Cs_8_Sn_46_ structure, (**c**) calculated phonon band structure along the ΧΖ-Ζ-Γ-X-XY-Γ path, and (**d**) partial phonon density of states for Cs and Sn of the Cs_8_Sn_44_ (model C).

We have conducted DFT calculations to investigate the phonon properties of the examined structures. We present the phonon spectrum, including the phonon band structure and phonon density of states (pDOS) of Cs_8_Sn_46−x_ (x = 0, 2). In Figure 8, we present the calculations for the phonon band structure and phonon DOS of the Cs_8_Sn_46_ compound and compare them with the phonon band structure and phonon DOS of the Cs_8_Sn_44_ structure (model C). The phonon bands are in good agreement with previous experimental and computational studies [22,44,45]. Additionally, we investigate the theoretical properties of the proposed (NH_4_)_8_Sn_44_ compound. The absence of imaginary frequencies in the phonon band structure confirms that the investigated materials are dynamically stable at 0 K. In the case of Cs_8_Sn_46_ (Figure 8a,b), we observe that several flat bands are introduced both in the acoustic and optical regions of the band structure, and that these are in good agreement with previous computational and experimental findings. As can be seen in pDOS plot, three distinct phonon bands resulting from Cs appear at 23 cm^−1^, 35 cm^−1^, and 50 cm^−1^, which can be attributed to the phonon modes of Cs atoms in the two different Sn cages of the material. It can be also seen that there are two regions with high density of states located above the acoustic modes and another near the top of the optical modes. For Cs_8_Sn_44_ (Figure 8c,d), the overall appearance of the phonon band structure and the pDOS are quite similar to the Cs_8_Sn_46_ with some distinctions. In the band structure diagram, we observe that the optical modes of Cs_8_Sn_44_ reach larger frequencies with respect to Cs_8_Sn_46_ analogue. As seen in the pDOS plot, these optical modes are related to the Sn atoms of the framework and their appearance can be attributed to the existence of three-coordinated Sn atoms in the framework. The number of localized bands in the band diagram are further increased for the same reason. For the phonon modes related to the Cs atoms, we can observe a shift of the low frequency peak in higher frequencies and a broadening of the two peaks in the region of 30 cm^−1^ to 60 cm^−1^ owing to the small distortions of the Sn cages and the reduction of framework symmetry due to the creation of Sn vacancies.

**Figure 9:**
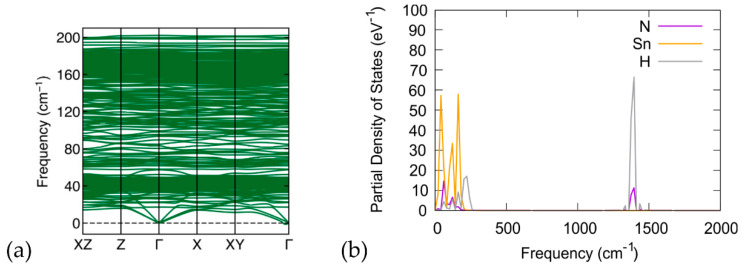
(**a**) Calculated phonon band structure along the ΧΖ-Ζ-Γ-X-XY-Γ path and (**b**) partial phonon density of states for N, H, and Sn atoms for the (NH_4_)_8_Sn_44_ (model C).

We also present the (NH_4_)_8_Sn_44_ (model C) calculations of the phonon plots (Figure 9a) and phonon density of states (Figure 9b). The NH_4_ cation produces increased phonon bands compared to Cs_8_Sn_44_, which is expected as a result of the large number of rotational and stretching modes of the ammonium cation (e.g., in NH_4_Cl [46]). Apart from the low frequency modes, vibrational modes of the ammonium cation at 1380 cm^−1^ and ca. 3000 cm^−1^ are found.

## 4. Discussion

Our computational study shows that the most energetically favored distribution of the Sn defects in the crystal structure of Cs_8_Sn_44_ is model C, with one defect per six-membered ring in the 3D spiro-network of Sn framework atoms. This is in good agreement with the literature for A_8_Sn_44_ [20]. It is worth noting that a fully ordered configuration has not been experimentally confirmed even at temperatures close to 0 K via synchrotron powder diffraction [21]. This analysis also led us to a reliable initial model for the hypothetical (NH_4_)_8_Sn_44_. As with Cs_8_Sn_44_, the (NH_4_)_8_Sn_44_ model C is the most stable and shows semiconducting behavior.

The use of ammonium cations instead of cesium may trigger a new synthetic approach based on reactive precursors and ammonium salts. The only experimental outcome relevant to our current study concerning the presence of a molecular guest in semiconducting clathrates is the clathrate-I Na_5.5_(H_2_)_2.15_Si_46_, which is stable at ambient temperature and pressure [47] and is prepared by mixing the Zintl phase NaSi and NH_4_Br under dynamic vacuum at 573 K. The presence of H_2_ seems to stabilize the framework, as no ‘empty’ clathrate-I Si_46_ can be formed under these or any other conditions. Except for H_2_, no neutral guests have been reported so far in semiconducting clathrates. This is attributed to the limited chemical affinity of the neutral molecules with an inorganic framework. Only one theoretical study on the use of Xe gas as guest has been performed, and that study suggests the incorporation under specific conditions in type-II Sn clathrate Xe_24_[Sn_136_] [48]. On the other hand, ammonia molecules as neutral guests are known in clathrates hydrates and they stabilize the framework, e.g., type-II tetrahydrofuran + NH_3_ water clathrate [49].

With regard to thermoelectric efficiency, there are only limited experimental data on the A_8_Sn_44_ system that show a low performance compared to the current record in type-I clathrates, namely Ba_8_Ga_16_Ge_30_ that reaches a ZT of ca. 1 at 800 K [4]. The current analysis on the distribution of defects is of significance for the elucidation of the phonon scattering mechanism in this system.

## 5. Conclusions

The current work describes in detail the defect distribution in the Cs_8_Sn_44_ type-I clathrates and shows how it hinders the presence of the Sn defects exclusively in spiro-connected six-membered rings as the most stable configuration. It also suggests that the incorporation of ammonium as a light, molecular cationic guest in the polyhedral cavities is thermodynamically possible. Both Cs_8_Sn_44_ and (NH_4_)_8_Sn_44_ materials behave as narrow band gap semiconductors. Due to the strong vibration of the ammonium cation in the framework cavities, phonon scattering is achieved. Given the high cost and hazards when handling alkali metals, this study may also trigger the exploration of new members in this class of compounds with molecular cations and ultimately improve the thermoelectric properties in A_8_Sn_44_ clathrates. New synthetic attempts with the use of reactive precursors are currently in progress in our research group.

## Figures and Tables

**Table 1:** Chemical composition, closest distance between framework vacancies, and lattice parameters for the models of Cs_8_Sn_46−x_ and (NH_4_)_8_Sn_46−x_ as well as the calculated enthalpies of formation based on DFT calculations. For reference, the experimentally determined lattice for Cs_8_Sn_44_ is *a* = *b* = *c* = 12.107 Å [21], namely half of the 2 × 2 × 2 supercell (space group *Ia*-3*d*, No. 230).

Compound	Closest Distance between Vacancies (Å)	a (Å)	b (Å)	c (Å)	Volume (Å^3^)	Vacancy E_formation_ (eV)	Compound E_formation_ (eV)
Cs_8_Sn_46_	−	12.497	12.497	12.497	1951.75	−	−8.948
Cs_8_Sn_44_ (model A)	8.154	12.100	12.135	12.137	1782.13	−0.411	−9.359
Cs_8_Sn_44_ (model B)	6.165	12.176	12.101	12.122	1785.98	0.125	−8.832
Cs_8_Sn_44_ (model C)	7.407	12.157	12.069	12.157	1783.71	−0.555	−9.503
(NH_4_)_8_Sn_46_	−	12.384	12.406	12.406	1906.062	−	−6.689
(NH_4_)_8_Sn_44_ (model A)	8.154	12.016	12.044	12.051	1743.993	−0.5063	−7.195
(NH_4_)_8_Sn_44_ (model B)	6.165	12.001	11.982	12.255	1760.361	0.3164	−6.372
(NH_4_)_8_Sn_44_ (model C)	7.407	12.083	11.957	12.075	1744.555	−0.9322	−7.621

## Data Availability

Dataset available on request from the authors.

## Supplementary Materials

The following supporting information can be downloaded at: https://www.mdpi.com/article/10.3390/ma17184595/s1, Figure S1: partial DOS of Cs_8_Sn_46_.; Figure S2: partial DOS of Cs_8_Sn_44_ (model C); Figure S3: partial DOS of (NH_4_)_8_Sn_46_, Figure S4: partial DOS of (NH_4_)_8_Sn_44_ (model C).
