# Supplementary material for: A DFT Computational Study of Type-I Clathrates A8Sn46−x (A = Cs or NH4, x = 0 or 2)
Source: Materials (Basel). 2024 Sep 19;17(18):4595. doi: 10.3390/ma17184595 (PMC11433220; doi:10.3390/ma17184595)
Supplement: Supplementary file 1 [file materials-17-04595-s001.zip › materials-3165135-supplementary.pdf]

Supporting Information

A DFT computational study of type-I clathrates A_8Sn_{46-x} ($A = Cs$ or NH_4, $x = 0$ or 2)

Nikolaos Kelaidis, Emmanuel Klontzas, Andreas Kaltzoglou*

Theoretical and Physical Chemistry Institute, National Hellenic Research Foundation, 11635, Athens, Greece

* Correspondence: akaltzoglou@eie.gr ; Tel.: +30 210 7273845

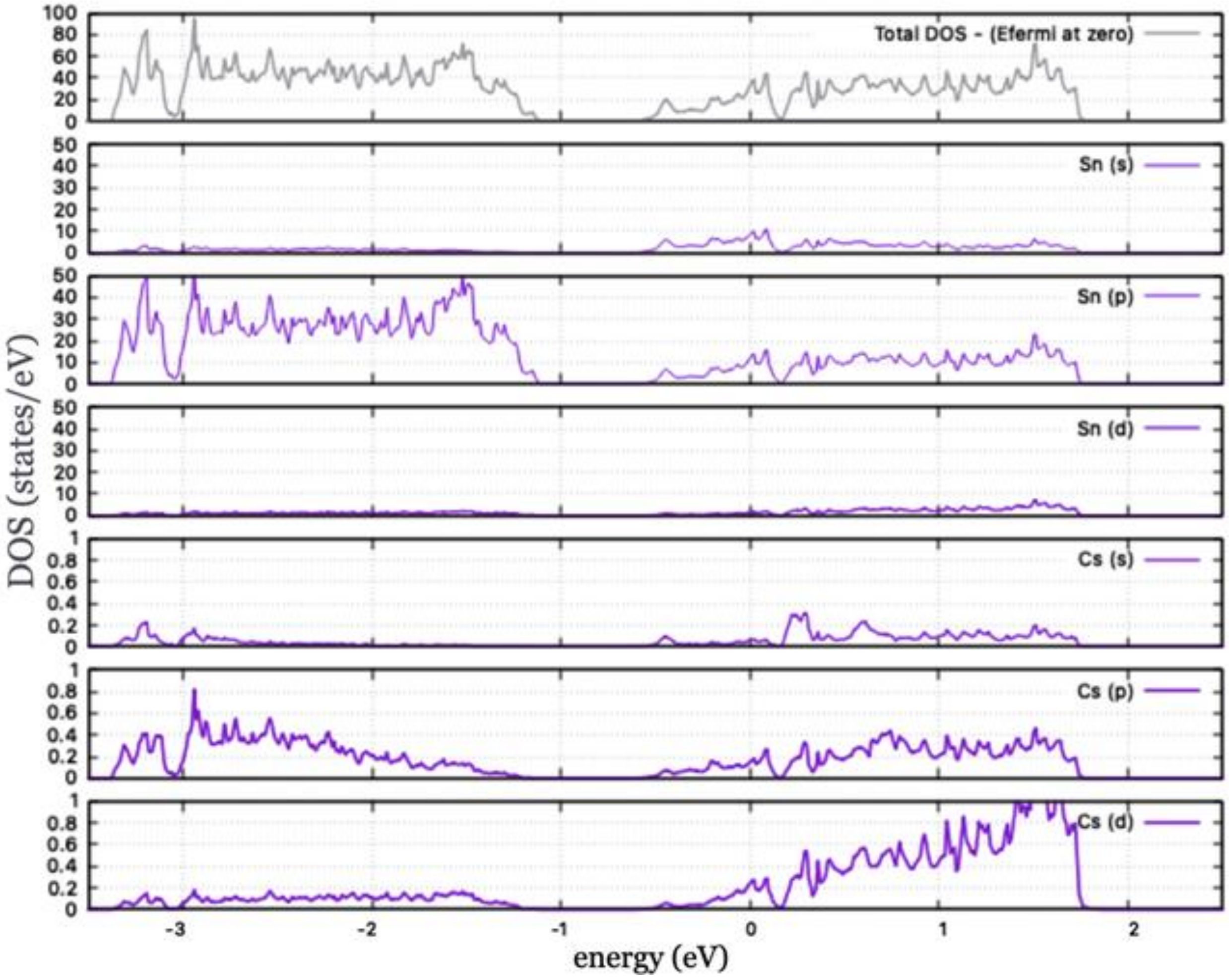

Figure S1. Partial DOS of Cs_8Sn_{46}.

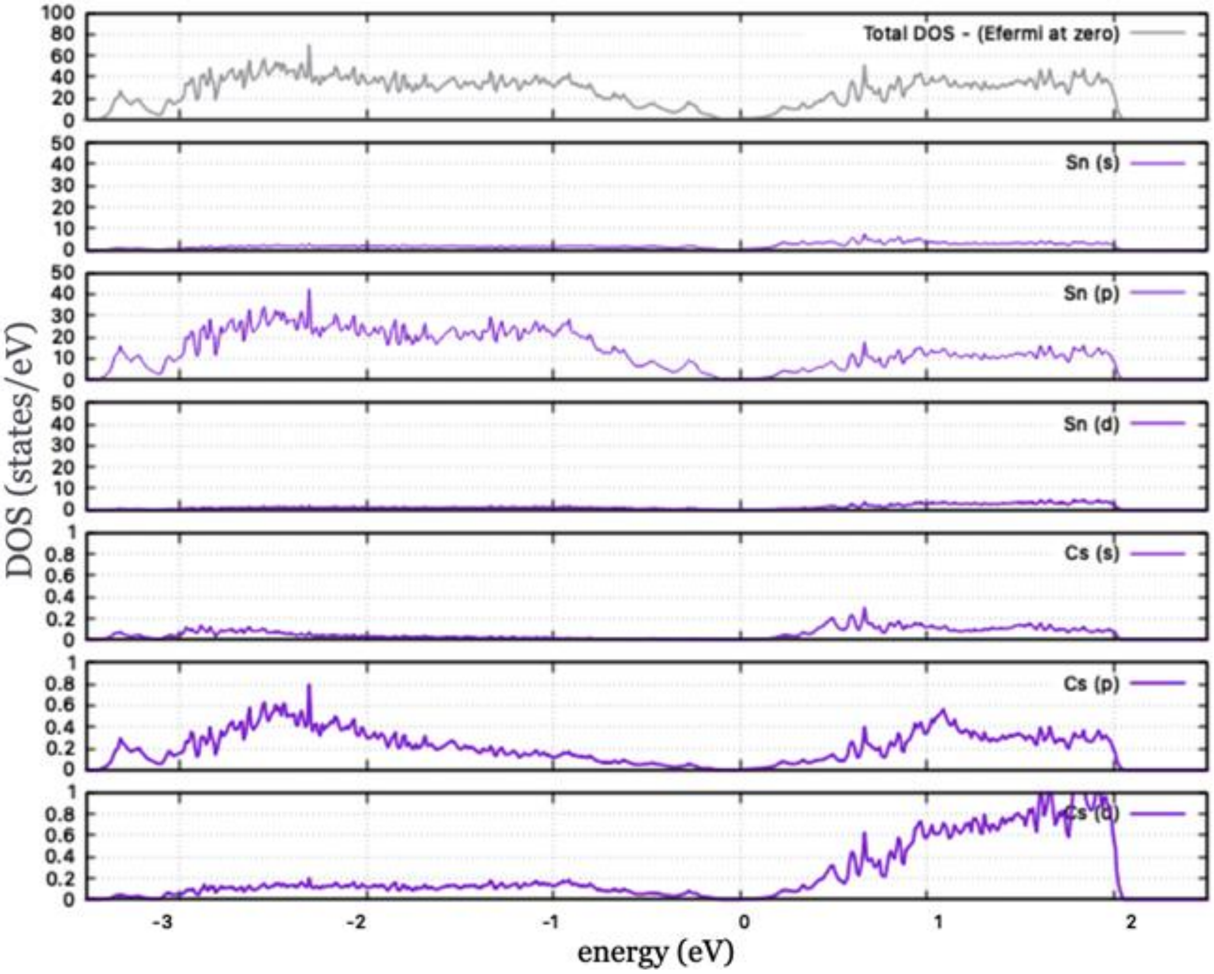

Figure S2. Partial DOS of Cs_8Sn_{44} (model C).

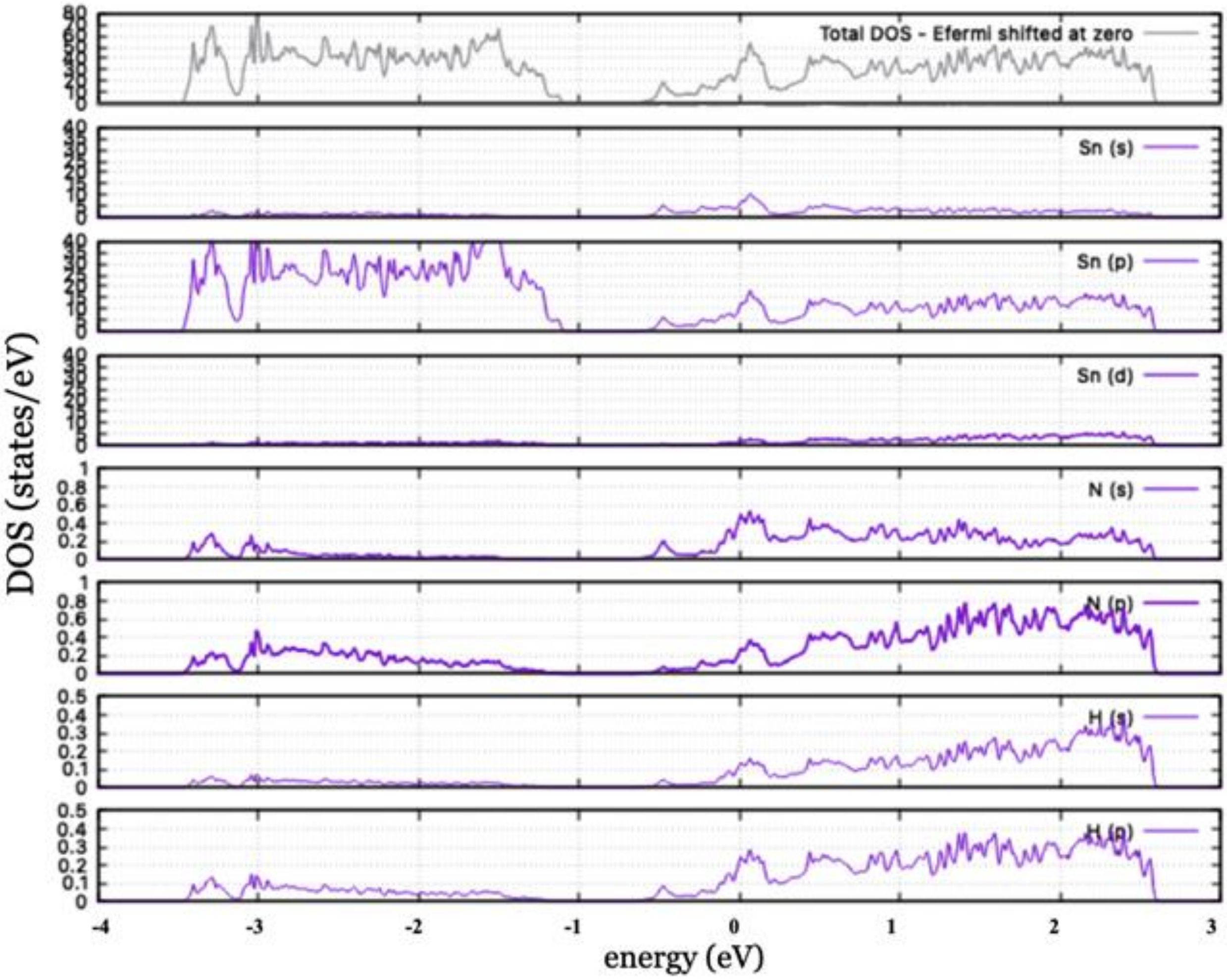

Figure S3. Partial DOS of $(NH_4)_8Sn_{46}$.

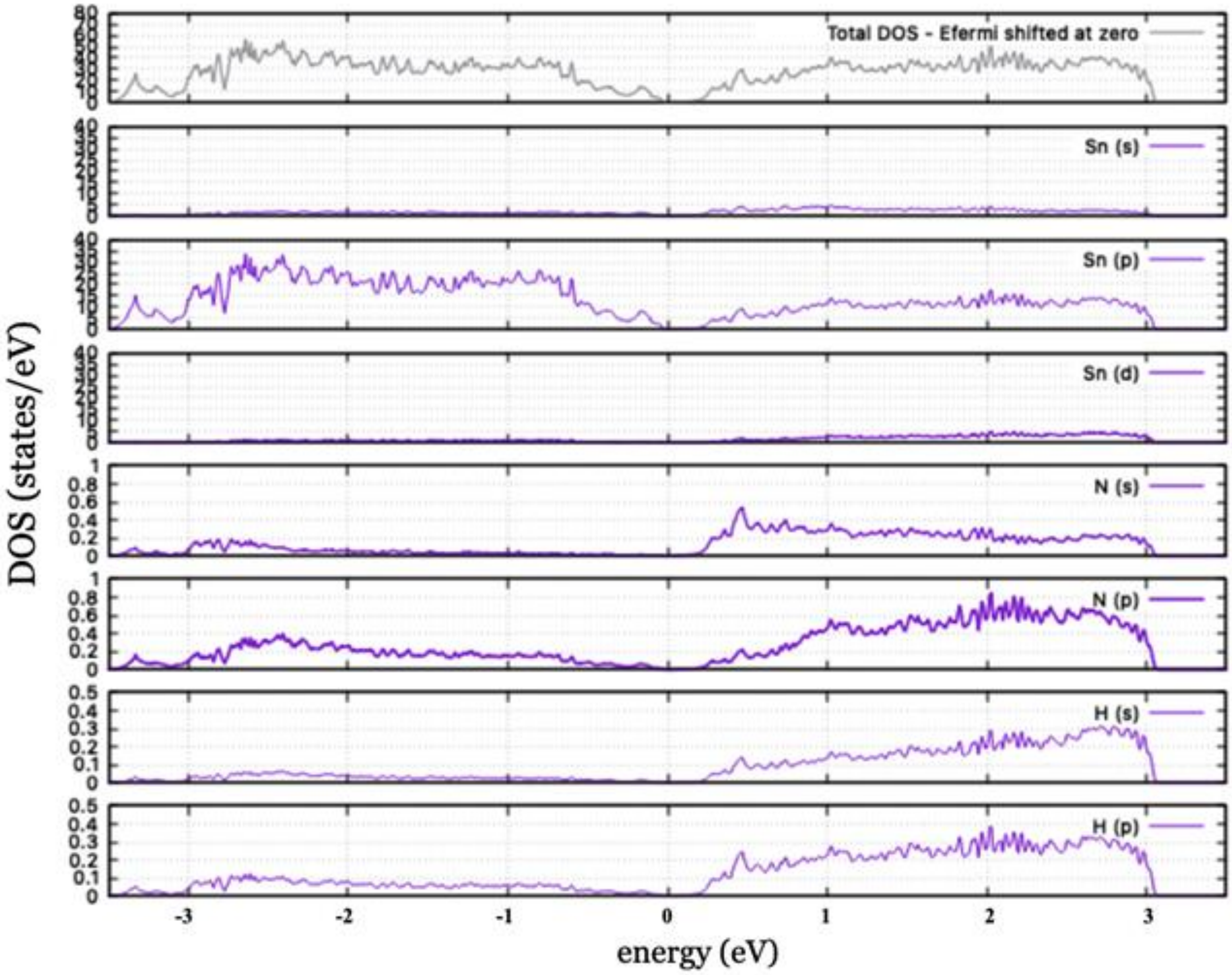

Figure S4. Partial DOS of $(NH_4)_8Sn_{44}$ (model C).
